# Oxygen Content-Controllable Synthesis of Non-Stoichiometric Silicon Suboxide Nanoparticles by Electrochemical Anodization

**DOI:** 10.3390/nano10112137

**Published:** 2020-10-27

**Authors:** Jaewoo Lee, Sang Yoon Lee, Heon Yong Jeong, Sung Oh Cho

**Affiliations:** Department of Nuclear and Quantum Engineering, Korea Advanced Institute of Science and Technology (KAIST), Daejeon 34141, Korea; jw.lee@kaist.ac.kr (J.L.); sangyoonlee@kaist.ac.kr (S.Y.L.); jeong93@kaist.ac.kr (H.Y.J.)

**Keywords:** nanoparticle, silicon suboxide, anodization, oxygen content

## Abstract

A facile route to producing non-stoichiometric silicon suboxide nanoparticles (SiO_x_ NPs, 0 < x < 1) with an adjustable oxygen content is proposed. The process is based on electrochemical anodization involving the application of a strong electric field near the surface of a Si electrode to directly convert the Si electrode into SiO_x_ NPs. The difference in ion mobility between oxygen species (O^2−^ and OH^−^), formed during anodization, causes the production of non-stoichiometric SiO_x_ on the surface of the Si while, simultaneously, fluoride ions in the electrolyte solution etch the formed SiO_x_ layer, generating NPs under the intense electric field. The adjustment of the applied voltage and anodization temperature alters the oxygen content and the size of the SiO_x_ NPs, respectively, allowing the characteristics of the NPs to be readily controlled. The proposed approach can be applied for mass production of SiO_x_ NPs and is highly promising in the field of batteries and optoelectronics.

## 1. Introduction

Nanoparticles are highly valuable for an extensive range of applications due to their high specific surface area. Silicon suboxide nanoparticles (SiO_x_ NPs, 0 < x < 1), in particular, exhibit unique electrical, optical, chemical, and mechanical properties, allowing the particles to be actively used as semiconductors and a coating material [1,2,3]. The properties of SiO_x_ NPs are heavily dependent on the oxidation state of the Si [4,5,6], which determines the oxygen content of the NPs.

SiO_x_ NPs are being studied largely for their application as the anode material in lithium-ion batteries to potentially meet the high demand for batteries with higher capacity and longer service life [1,4,7,8,9]. Currently, the batteries are reported to have a high charge capacity [2,10,11,12] and a low volume expansion ratio [8,13]. Lithium oxides (e.g., Li_2_O and Li_4_SiO_4_), produced by Li ion insertion into SiO_x_ during initial charging, serve to buffer volume changes, improving charge cycle performance [14,15]. It has been previously reported that the charge capacity and coulombic efficiency increase while the cyclability of the batteries decreases as the oxygen content of SiO_x_ NPs decreases [4,8,10,13]. Therefore, the optimal oxygen content of SiO_x_ NPs must be considered in order to produce efficient lithium-ion batteries.

SiO_x_ NPs are also commonly applied as anti-reflective coating due to their attractive optical properties [16,17]. Many studies have found that the refractive index of SiO_x_ decreases as the oxygen content increases [3,18]. Therefore, being able to alter the oxygen content of SiO_x_ NPs allows the refractive index of the NPs to be controllable and, with adjustable refractive index, applications of SiO_x_ NPs are diverse in the field of optics. Furthermore, the anti-reflectivity of SiO_x_ NPs is not limited to a particular wavelength; the NPs can improve the photovoltaic efficiency at various wavelengths [6,19].

The excellent passivating property of SiO_x_ NPs also makes the NPs suitable for incorporation into organic light-emitting diodes [3,6,20]. SiO_x_ NPs are chemically and mechanically stable in general, but by adjusting the oxygen content of the NPs, the reactivity of the NPs can be increased. In addition, SiO_x_ NPs have abundant oxygen vacancies, allowing the NPs to be potentially utilized as a sensing material [21,22,23]. Oxygen vacancies become more prevalent and electrical conductivity increases as the oxygen content of SiO_x_ NPs decreases [24,25]. Both the passivating and insulating properties of SiO_x_ play an important role in determining the sensitivity and selectivity of gas sensing, thus, synthesizing SiO_x_ NPs with adjustable oxygen content is highly important.

Si is not very reactive at low temperatures; therefore, conventional SiO_x_ NPs synthesis procedures involve high-temperature reactions. The high-temperature reactions can be classified into two categories: melting-condensation [9] and chemical vapor deposition [1,2,4]. In the former, Si (or Si mixed with silica) is melted and oxygen is injected. The formed molten SiO_x_ is then evaporated, after which, the vaporized SiO_x_ is condensed rapidly and extracted in the form of NPs. In the chemical vapor deposition reaction, microwave is used to induce the plasma state of Si-based precursors, allowing oxygen to be deposited into Si. Non-stoichiometric SiO_x_ is created by altering the pressure and the amount of oxygen present during the reaction, and the volatile molecules are extracted as NPs. Nonetheless, both approaches have difficulties fabricating SiO_x_ particles smaller than 200 nm, are highly time-consuming, and require complicated setups for housing high-temperature systems.

In this paper, we propose the use of anodization, an electrochemical process, to synthesize SiO_x_ NPs with adjustable oxygen content. Anodization is typically employed to fabricate a uniform nanostructured (nanotubular or nanoporous) oxide layer on metallic surfaces [26,27,28,29]. The two key reactions required to form nanostructures are oxidation and etching. When a metal surface is oxidized, etching agents in the electrolyte react with the oxide layer to form nanostructures. A recent study reported that metal oxide NPs can be directly obtained from anodizing metal wires [30]. However, to the best of our knowledge, there have been no attempts to synthesize oxide NPs from anodizing poorly conductive metalloids like Si. In addition, unlike the previously synthesized metal-based NPs (e.g., Al_2_O_3_, TiO_2_, ZrO_2_, etc.), the SiO_x_ NPs synthesized in this work are non-stoichiometric. The anodization process provides multiple advantages, such as immediacy, simplicity, capability to synthesize at low temperatures, and economic efficiency, over the conventional procedures. In this study, we have successfully synthesized SiO_x_ NPs with adjustable oxygen content and size, and the NPs will have significant applications in the fields of batteries and optoelectronics.

## 2. Materials and Methods

### 2.1. Materials

Doped Si is conductive enough to allow for anodization. P-type Si is generally used, but n-type Si can also be employed under specific illumination conditions [31,32]. To simplify the experimental process, boron-doped p-type Si wafers (<100>) with a resistance of 0.001–0.003 Ω cm (Tasco, Miami, FL, USA) were used in this work. In order to generate a very intense electric field adjacent to Si, the wafers were cut into 1.0 mm × 0.5 mm thin rods with a high aspect ratio by arc discharging in water. Halogen elements are generally used as etching agents, thus, in this study, fluorine anions from reagent-grade ammonium fluoride (NH_4_F) (Sigma-Aldrich, St. Louis, MO, USA) were chosen. The electrolyte solution was prepared with deionized (DI) water.

### 2.2. SiO_x_ NPs Preparation

**Figure 1:**
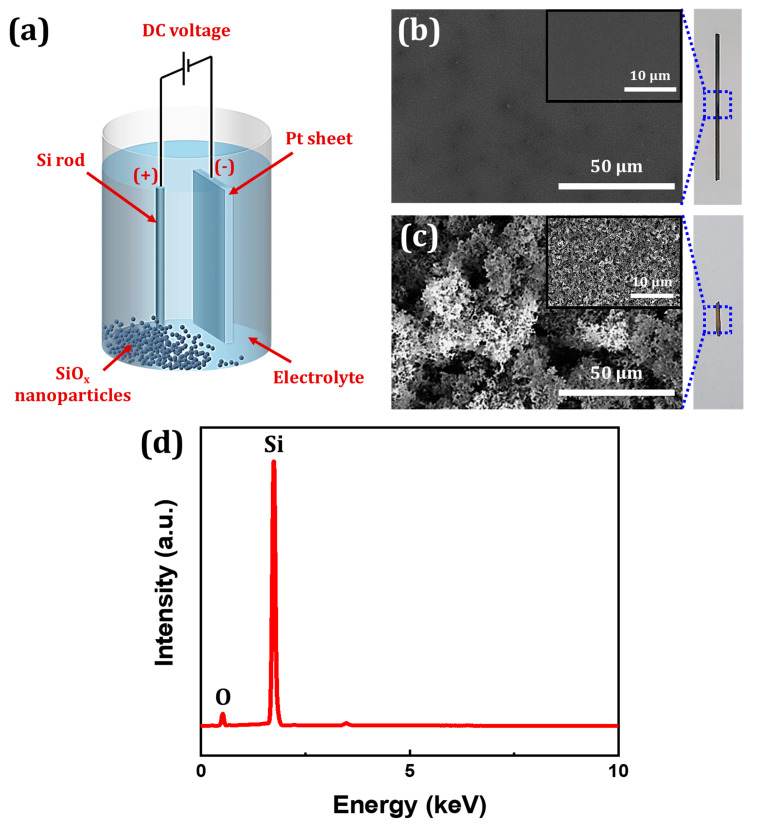
(**a**) An illustration showing the two-electrode anodization process for SiO_x_ nanoparticle (NP) synthesis. FESEM images of a Si rod surface (**b**) before and (**c**) after anodization (digital images shown on the right). (**d**) EDX spectrum of the Si rod surface after anodization at a constant voltage and temperature of 7.5 V and 10 °C, respectively.

Prior to anodization, Si rods were sonicated in acetone and ethanol for 5 min each, followed by rinsing with DI water to remove any impurities present on the surfaces of the rods. The rods were then dried under a nitrogen stream and no further treatments were performed on the rods. Anodization was performed in 10 M NH_4_F aqueous solution with a typical two-electrode setup employing a Si rod as the anode and a platinum sheet (10 mm × 40 mm × 0.5 mm) as the cathode (Figure 1a). The two electrodes were placed 10 mm apart and a voltage was applied with a 900 W DC power supply (OPS-3003, ODA Technologies, Incheon, Korea). The anodization process was carried out at a temperature that was kept constant using a thermostatic bath (RW-3040G, Lab. Companion, Daejeon, Korea) and was maintained for 1 h. During anodization, NPs could be observed precipitating. After anodization, the NPs were extracted by vacuum filtration. The NPs were then rinsed thoroughly with DI water and dried in a desiccator at room temperature under vacuum.

### 2.3. Characterization

The synthesized NPs and the surface morphology of a Si rod before and after anodization were characterized using a field emission scanning electron microscope (FESEM, Magellan400, FEI, Hillsboro, OR, USA). The size of the NPs was determined from FESEM images collectively containing at least 100 particles utilizing the ImageJ software (Laboratory for Optical and Computational Instrumentation, University of Wisconsin-Madison, Madison, WI, USA) [33]. The elemental composition of the anodized Si rod surface was deduced using an energy-dispersive X-ray spectrometer (EDX) equipped with the FESEM. Further investigation into the morphology and crystallinity of the NPs were carried out with a transmission electron microscope (TEM, Titan cubed G2 60-300, FEI, Hillsboro, OR, USA). Chemical bonds present in the NPs were analyzed with an X-ray photoelectron spectrometer (XPS, K-alpha, Thermo VG Scientific, Waltham, MA, USA) for Al Kα radiation (1486.7 eV).

## 3. Results and Discussion

### 3.1. Synthesis of Silicon Oxide NPs

**Figure 2:**
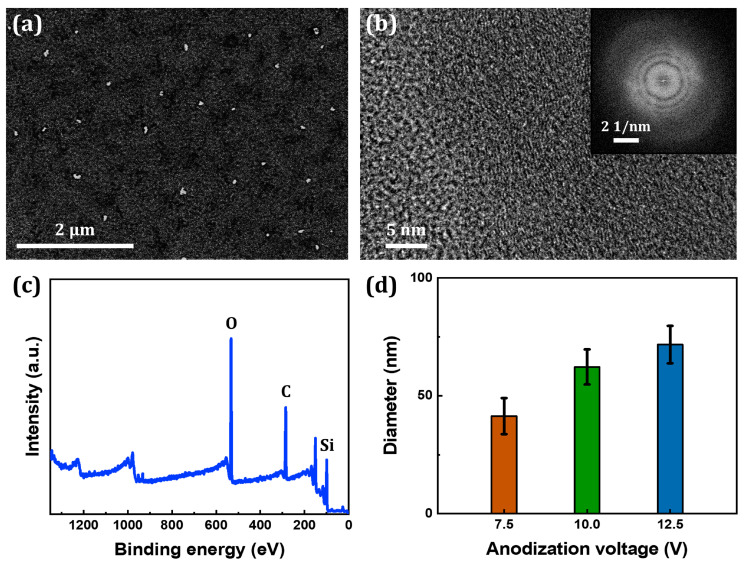
(**a**) FESEM image, (**b**) high-resolution TEM image (inset: SAED pattern), and (**c**) XPS survey spectrum of SiO_x_ NPs synthesized at a temperature of 5 °C and a constant voltage of 10.0 V. (**d**) Average particle size of synthesized SiO_x_ NPs as a function of anodization voltage.

Electrochemical anodization was performed on a Si rod at a constant voltage and temperature of 7.5 V and 10 °C, respectively. After completion of the process, the surface of the Si rod was examined for any changes. It was visibly noticeable that the rod became shorter and lost its luster due to etching and oxidation, respectively (digital photographs shown beside Figure 1b,c). Figure 1b,c shows FESEM images of the surface of the Si rod before and after anodization. The initially smooth surface of the rod became highly rough after anodization. EDX spectrum, shown in Figure 1d, confirms that the roughness indeed arises from the silicon oxide layer that has formed on the surface of the rod. The anodization experiment was then repeated, but at different voltages of 7.5, 10.0, and 12.5 V. The temperature at which the process was carried out was also set to 5 °C to mitigate the local heat buildup resulting from the high electrical resistance of Si. In all cases, precipitation was observed; Figure 2a shows that the formed precipitate consists of individual particles with a size of less than 100 nm. The intense electric field near the Si surface allows the silicon oxide layer to be extracted as NPs. The obtained FESEM image was adjusted to have high contrast for size distribution analysis. Each particle was assumed to be spherical and the diameter of each particle was calculated using ImageJ. From the high contrast image, as shown in Figure S1, it was determined that the NPs were approximately 41.4, 62.2, and 71.8 nm in diameter with only a slight variance when synthesized at constant voltages of 7.5, 10.0, and 12.5 V, respectively (Figure 2d). A possible explanation for this phenomenon is that the local electrolyte temperature changes as the applied voltage varies due to Joule heating becoming more prevalent as the voltage rises. Investigation into the crystallinity of particles suggests that the particles have no specific crystallographic orientation as shown in the high-resolution TEM image (Figure 2b). The selected area electron diffraction (SAED) pattern, shown in the inset of Figure 2b, further confirms that the particles are amorphous. The XPS measurement of the synthesized particles was collected to identify the elements present; prominent Si and O peaks can be observed in the XPS spectrum (Figure 2c). The wt% of Si and O vary slightly with the applied voltage (Table S1). The result supports that the particles are SiO_x_. The observable carbon peaks near 285 eV can be attributed to the membrane filter utilized to extract the particles and the copper tape used during the XPS measurement, and the sharp peak near 150 eV can be attributed to plasmons [34]. No peaks corresponding to the presence of nitrogen and fluorine, two elements found in the electrolyte, can be observed. From these results, it can be concluded that SiO_x_ NPs can be directly synthesized via anodization.

### 3.2. Control of Oxygen Content in SiO_x_ NPs

The oxygen content of SiO_x_ can be described as the average oxidation number of Si. The oxidation number is expressed as the x-value in “SiO_x_”. The x-value has a significant effect on the properties of SiO_x_. In Si, the Si atoms are arranged in tetragonal formations with four atoms surrounding an atom. When Si is oxidized, the four surrounding atoms are replaced with O atoms. The number of replacements that occur determines the oxidation number of Si, and this number can range from 0 to 4; the possible resulting states are Si-(Si_4_), Si-(Si_3_O), Si-(Si_2_O_2_), Si-(SiO_3_), and Si-(O_4_). The binding energy varies slightly for each oxidation state. According to the random-bonding model, SiO_x_ is a combination of the five states and is amorphous. In this connected SiO_x_ state, Si-(Si_3_O), Si-(Si_2_O_2_), Si-(SiO_3_), and Si-(O_4_) can be expressed as Si_2_O, SiO, Si_2_O_3_, and SiO_2_, respectively. The frequency distribution of the five oxidation states determines the oxidation number, meaning that the x-value can be adjusted by carefully controlling the production of each oxidation state. Through Si 2p scanning, the x-value can be determined employing the equation given in Equation (1).

(1) $$x=\frac{0.0\times a+0.5\times b+1.0\times c+1.5\times d+2.0\times e}{a+b+c+d+e},$$

where *a*, *b*, *c*, *d*, and *e* are the peak areas of Si^0^, Si^1+^, Si^2+^, Si^3+^, and Si^4+^ peaks, respectively. There are two distinguishable Si^0^ peaks resulting from spin-orbital splitting into Si 2p_1/2_ and Si 2p_3/2_; the peak ratio between these two peaks is approximately 1:2, respectively [34]. Table 1 provides Si 2p peak information for each oxidation state. The binding energy of each peak varies slightly with the x-value, but the variance is insignificant [35].

**Figure 3:**
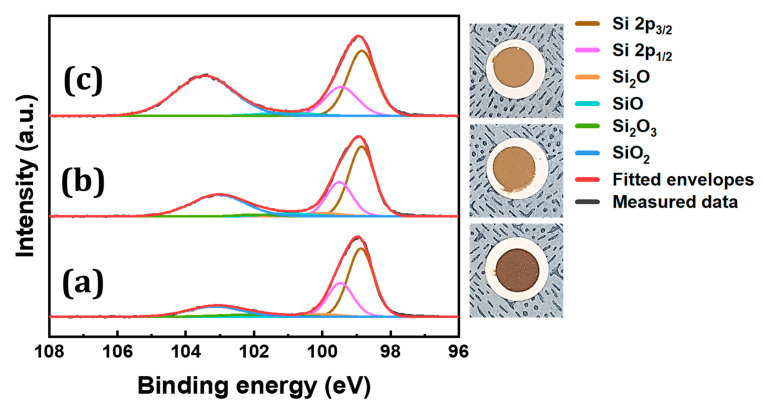
XPS spectra and digital photographs of SiO_x_ NPs synthesized at voltages of (**a**) 7.5 V, (**b**) 10.0 V, and (**c**) 12.5 V at a constant temperature of 5 °C.

Si 2p spectra, shown in Figure 3, visualizes the chemical-bond distributions of SiO_x_ NPs synthesized at different anodization voltages. All spectra were referred to the C 1s line (285 eV). Six peaks can be found in each spectrum with the highest peaks located near 99 and 103 eV, the former of which corresponds to the amalgamation of Si 2p_1/2_ and Si 2p_3/2_ peaks. The presence of these two prominent peaks are attributed to Si–Si bonding and O–Si–O bonding. Much less noticeable peaks can be found near 100, 101, and 102 eV, indicating relatively little formation of Si_2_O, SiO, and Si_2_O_3_. Thus, the result supports that the most frequent oxidation state present in the NPs is the +4 (SiO_2_) state, excluding the zero-oxidation state. This uneven formation of oxidation states can be explained as SiO_2_ being the most stable oxidation state. Furthermore, as anodization voltage increases, SiO_2_ peak becomes even more dominant. The x-values of SiO_x_ synthesized at voltages of 7.5, 10.0, and 12.5 V were calculated to be 0.42 ± 0.05, 0.64 ± 0.09, and 0.89 ± 0.09, respectively, indicating that the oxygen content of SiO_x_ NPs increases with rising anodization voltage.

**Figure 4:**
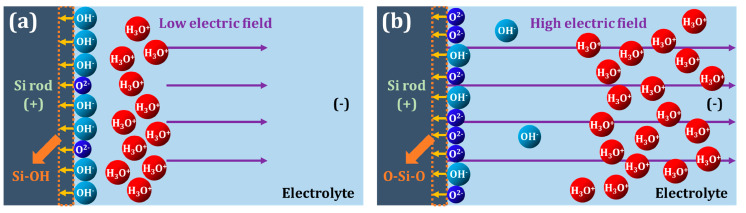
Illustrations of how oxygen content is affected by the anodization voltage ((**a**) low voltage and (**b**) high voltage).

The mechanism behind the oxygen content change resulting from anodization voltage adjustment is visualized in Figure 4. During anodization, anions of oxygen species (O^2−^ and OH^−^) are created by field-assisted deprotonation of water [26,39,40]. It is generally accepted that ion mobility greatly affects the oxidation process during anodization [41,42]. Ion mobility, in a solution, is directly proportional to the charge of the ion and inversely proportional to the mass of the ion. Thus, O^2−^ ions are transported much more quickly than OH^−^ under a constant electric field. This difference in mobility between the two anions leads to the observed variations in oxygen content of SiO_x_. As depicted in Figure 4a, at a low voltage, the low electric field produces little O^2−^, while also allowing the formed anions to recombine with H_3_O^+^. In addition, there is not a significant difference in mobility between the two aforementioned anions under low electric field. These two factors permit OH^−^ to readily bond with Si without having to compete with O^2−^, leading to the formation of Si–OH bonds, which are very soluble in the aqueous solution. Figure 4b shows that as the voltage rises, the electric field increases, producing more anions and increasing the mobility difference between O^2−^ and OH^−^. With the higher mobility, O^2−^ outcompetes OH^−^ and O–Si–O bonds become more dominant (SiO_2_ in Figure 3c).

### 3.3. Size Control of SiO_x_ NPs

**Figure 5:**
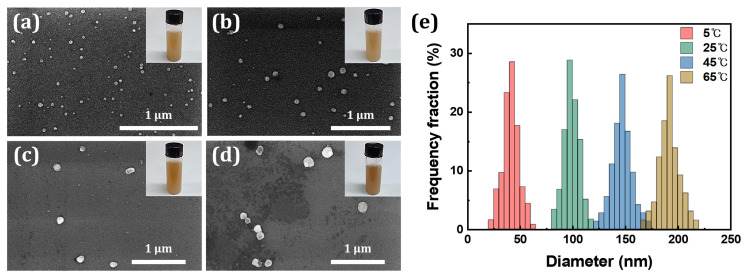
FESEM images of NPs synthesized at temperatures of (**a**) 5 °C, (**b**) 25 °C, (**c**) 45 °C, and (**d**) 65 °C at a constant voltage of 7.5 V (insets: digital photographs of synthesized SiO_x_ NPs dispersed in DI water). (**e**) The size distributions of synthesized NPs.

When SiO_x_ NPs were synthesized at a voltage and temperature of 7.5 V and 5 °C, respectively, the particles were on average 41.4 nm in diameter. Fabrication at higher temperatures increases the size of the synthesized NPs as shown in Figure 5; when fabricated at the temperatures of 25, 45, and 65 °C, the average sizes of the synthesized NPs were 99.2, 147.0, and 192.3 nm, respectively. Additionally, more spherical NPs were observed as the fabrication temperature rose. The x-value, however, was generally unaffected by the temperature.

Anodization temperature is typically one of the most important factors regarding the morphology of the synthesized oxides. When anodization occurs at a high temperature, the aqueous electrolyte solution has a low viscosity, allowing a high diffusion of F^−^, an etching agent [43]. Consequently, a high etching rate leads to a high oxidation rate [28,43], ultimately resulting in a production of large particles. Thus, through the adjustment of the anodization temperature, the size of the synthesized SiO_x_ NPs can be controlled.

## 4. Conclusions

Controlling the oxygen content of SiO_x_ NPs allows the NPs to exhibit diverse properties. We successfully synthesized non-stoichiometric SiO_x_ NPs by anodizing a doped Si rod in an aqueous electrolyte solution. A silicon oxide layer formed on the surface of the rod after anodization, indicating that oxidation and etching took place, and the electric field induced near the surface led to the production of NPs. Raising the applied voltage increased the oxygen content of the synthesized SiO_x_ NPs, a phenomenon that can be attributed to the greater generation and higher mobility of O^2−^ in comparison to OH^−^ under a high electric field. Furthermore, a higher anodization temperature caused more diffusion of F^−^, subsequently increasing the etching rate and the size of the synthesized NPs significantly. The anodization process, described in this work, is much simpler than the conventional procedures and can be used to quickly mass-produce SiO_x_ NPs by implementing a large number of Si electrodes. 

## Figures and Tables

**Table 1:** Si 2p peak information for different oxidation states (0 to 4).

Oxidation State	Group	Binding Energy (eV) [34,36,37,38]
Si^0^	Si	98.9 (Si 2p_3/2_) and 99.5 (Si 2p_1/2_)
Si^1+^	Si_2_O	100.0
Si^2+^	SiO	101.0
Si^3+^	Si_2_O_3_	102.1
Si^4+^	SiO_2_	103.2

## Supplementary Materials

The following are available online at https://www.mdpi.com/2079-4991/10/11/2137/s1, Figure S1: FESEM images and their high contrast counterparts of SiO_x_ NPs synthesized at voltages of (a) 7.5 V, (b) 10.0 V, and (c) 12.5 V at a constant temperature of 5 °C, Table S1: Wt% of the elements in SiO_x_ NPs synthesized at voltages of (a) 7.5 V, (b) 10.0 V, and (c) 12.5 V at a constant temperature of 5 °C.
