# Supplementary material for: Oxygen Content-Controllable Synthesis of Non-Stoichiometric Silicon Suboxide Nanoparticles by Electrochemical Anodization
Source: Nanomaterials (Basel). 2020 Oct 27;10(11):2137. doi: 10.3390/nano10112137 (PMC7693619; doi:10.3390/nano10112137)
Supplement: Supplementary file 1 [file nanomaterials-10-02137-s001.pdf]

Supplementary Information

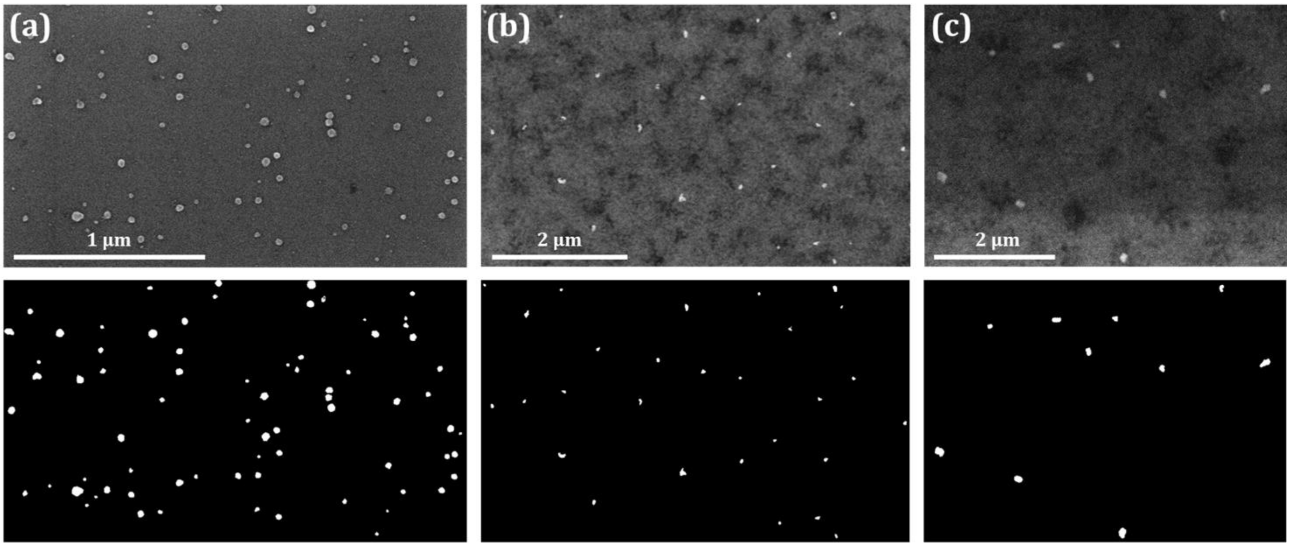

Figure S1. FESEM images and their high contrast counterparts of SiO_x NPs synthesized at voltages of (a) 7.5 V, (b) 10.0 V, and (c) 12.5 V at a constant temperature of 5 °C.

Table S1. Wt% of the elements in SiO_x NPs synthesized at voltages of (a) 7.5 V, (b) 10.0 V, and (c) 12.5 V at a constant temperature of 5 °C.

Anodization voltage (V)	Si (wt%)	O (wt%)
7.5	71.5	28.5
10.0	64.1	35.9
12.5	59.2	40.8
